# Controlling fluidic oscillator flow dynamics by elastic structure vibration

**DOI:** 10.1038/s41598-023-35643-1

**Published:** 2023-05-31

**Authors:** Innocentio A. Loe, Tianyi Zheng, Kiyoshi Kotani, Yasuhiko Jimbo

**Affiliations:** 1 Department of Precision Engineering, University of Tokyo, Tokyo, 113-0032 Japan; 2 Research Center for Advanced Science and Technology, University of Tokyo, Tokyo, 153-8904 Japan

**Keywords:** Fluid dynamics, Statistical physics, thermodynamics and nonlinear dynamics

## Abstract

In this study, we introduce a design of a feedback-type fluidic oscillator with elastic structures surrounding its feedback channel. By employing phase reduction theory, we extract the phase sensitivity function of the complex fluid–structure coupled system, which represents the system’s oscillatory characteristics. We show that the frequency of the oscillating flow inside the fluidic oscillator can be modulated by inducing synchronization with the weak periodic forcing from the elastic structure vibration. This design approach adds controllability to the fluidic oscillator, where conventionally, the intrinsic oscillatory characteristics of such device were highly determined by its geometry. The synchronization-induced control also changes the physical characteristics of the oscillatory fluid flow, which can be beneficial for practical applications, such as promoting better fluid mixing without changing the overall geometry of the device. Furthermore, by analyzing the phase sensitivity function, we demonstrate how the use of phase reduction theory gives good estimation of the synchronization condition with minimal number of experiments, allowing for a more efficient control design process. Finally, we show how an optimal control signal can be designed to reach the fastest time to synchronization.

## Introduction

Self-sustaining oscillation can be observed as a natural phenomenon in the study of fluid physics. Vortex shedding^1^ is one prime example of such phenomena, and is well-known to potentially cause flow-induced vibration^2^. On the contrary, fluidic oscillators are devices designed to generate oscillating flow which can be exploited for other uses. Ever since its invention, fluidic oscillators have found many uses such as flow separation control, drag reduction, flowmeter design, and fluid mixing^3–7^.

**Figure 1:**
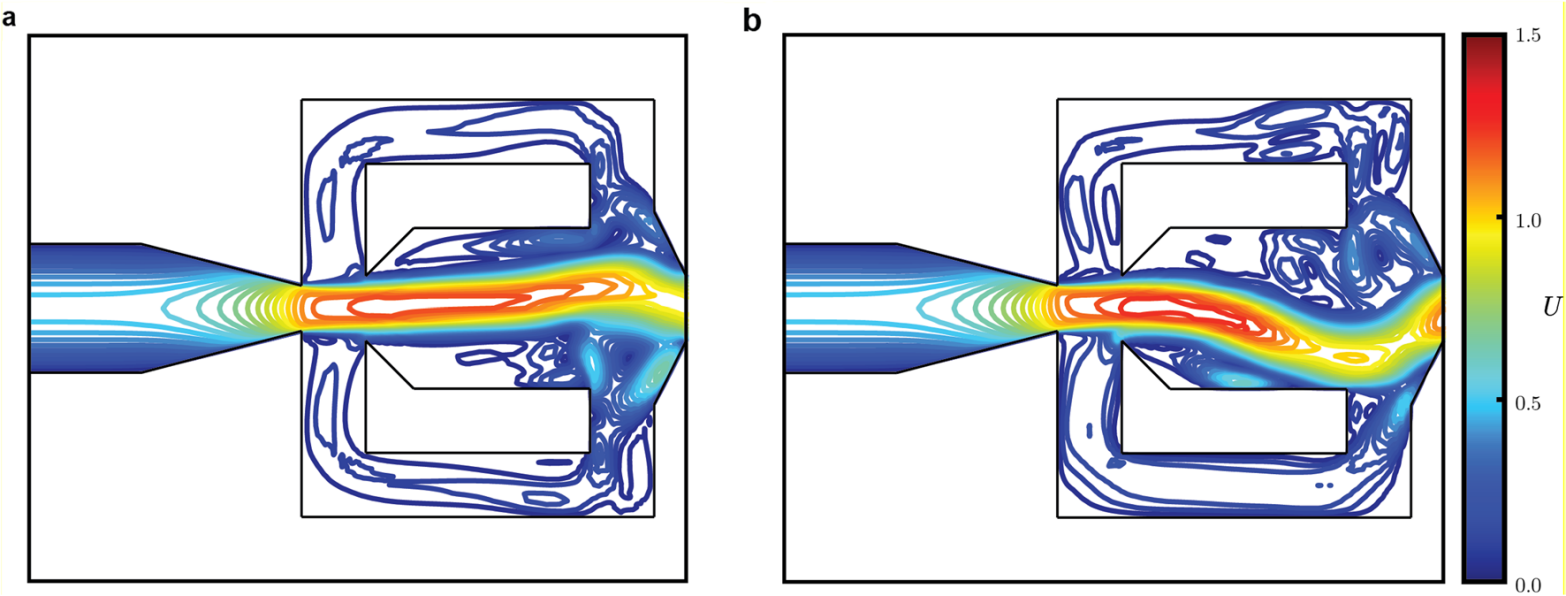
Mechanism of a typical feedback-type fluidic oscillator. The fluid flows through an inlet on the left side and exits through the outlet on the right side. Colored lines show the normalized velocity profile $U$. (**a**) Coanda effect causes a small portion of the fluid to return to the inlet area through the upper-side feedback channel. (**b**) The feedback flow from the upper-side feedback channel deflects the main inlet flow such that a portion of the fluid will now flow through the lower-side feedback channel. This process repeats alternately, creating a self-sustaining oscillation.

One of the most attractive characteristics of the fluidic oscillator is that it requires no moving component. In the case of the feedback-type fluidic oscillator, the self-sustaining oscillation is generated because the fluid jet supplied into the device will be attached to one side of the inner walls due to Coanda effect, causing a small fraction of the fluid jet to return through the feedback channel and deflect the main inlet flow to the other side. This process repeats alternately, creating a self-sustaining oscillation^8–10^, as illustrated in Fig. 1. However, this characteristic imposes a limitation in which the geometry of the device defines the resulting characteristics of the oscillatory flow, including its frequency^10,11^. The simplest method to modulate the oscillation frequency is to change the inlet pressure, which will consequently increase the inlet flow rate. However, an increase in inlet pressure may not be desirable as the device must be able to withstand larger pressure value^12^. Furthermore, the change in inlet flow rate may also change the flow regime from laminar to turbulent flow.

Mechanisms to generate oscillatory flow in a fluidic oscillator with frequency independent of its inlet flow condition has been considered in past studies. Methods that employ direct excitation such as by a piezoelectric actuator^13^ or a plasma discharge mechanism^14^ are common examples. However, the fluid flow itself does not intrinsically exhibit oscillatory dynamics in such cases. Other methods use the generated sweeping jet as additional feedback^9^, or use a cascaded oscillator geometry^15,16^.

In order to analyze a fluidic oscillator performance and its resulting fluid flow characteristics, a complicated experimental work is usually required, from manufacturing the device to setting up the equipment necessary for data acquisition, such as with hot-wire anemometry^3,15,16^. Numerical simulation allows calculation of the fluid flow characteristics of a fluidic oscillator design before the actual experiment. However, apart from conducting multiple simulations with different parameters, it is generally difficult to estimate the controllability of the system or to find optimal settings under given restrictions.

On the other hand, the use of reduced order models to describe oscillatory flow has become popular as it allows for a simplified description of the underlying physics, and predicts the resulting fluid flow characteristic for a given control input^17–21^. For oscillating flow control, periodic forcing to induce synchronization is a rigorously studied subject^21–23^. Applying model reduction theory will benefit this field by simplifying its analysis. Model reduction based on phase reduction theory was first studied by Taira and Nakao, who found that low-dimensional dynamic representation using a single phase equation could estimate synchronization properties of a vortex shedding past a cylinder^24^. While the method is more commonly used in studies of biological systems^25,26^, it has gained much interest in the fluid physics community in the recent years^22,27,28^. Other studies have expanded its usage to fluid–structure interactions^29^ and thermoacoustic systems^30^.

In this work, we propose a control mechanism to modulate oscillation frequency of a feedback-type fluidic oscillator by synchronization with weak external forcing in the form of vibration of elastic structures attached to the fluidic oscillator body. In our proposal, the intrinsic oscillatory flow is maintained even without external forcing, while the moving component adds controllability to the system. Phase reduction theory is used to simplify the analysis of the synchronization condition of the complex fluid–structure interaction dynamics, and allows estimation of the control region. We demonstrate how different placement of the elastic structure leads to a different phase response and analyze the change in fluid flow properties both for when synchronization is achieved or not. Our results show that the synchronization condition is well estimated using phase reduction theory, and that the synchronization induces changes in physical properties of the oscillating fluid motion. In addition, we demonstrate how an optimal control framework can be used to design the shape of the periodic forcing signal that allows for faster synchronization time.

## Results and discussion

### Oscillatory characteristics

**Figure 2:**
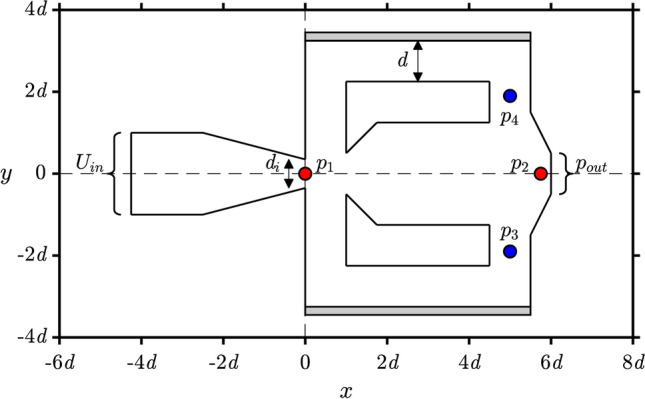
The fluidic oscillator model. ${p}_{1}$–${p}_{4}$ mark the measurement points of the pressure values to evaluate the oscillatory characteristics. ${U}_{\rm{in}}$ and ${p}_{\rm{out}}$ respectively denote the velocity inlet boundary condition and the pressure outlet boundary condition. The feedback channel width $d$ is used as the scaling factor, and the power nozzle width ${d}_{i}=0.7d$. The gray area around the feedback channel represents the elastic structure. See "Methods” section for more details.

**Figure 3:**
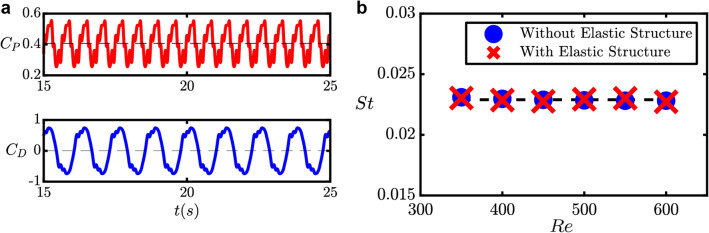
Oscillatory dynamics. (**a)** Time-course of the pressure loss coefficient ${C}_{P}$ and driving force coefficient ${C}_{D}$ when stable oscillation is achieved. (**b**) Comparison of the Strouhal number $St$ versus Reynolds number $Re$ with and without the addition of the elastic structure shows that the intrinsic dynamics of the oscillatory flow is not affected by the elastic structure.

We begin by describing the design of the fluidic oscillator model used in this study and observing its oscillatory characteristics. We use a typical feedback-type fluidic oscillator geometry in our simulation setup, as shown in Fig. 2. This geometry is adapted from a previous study by Seo et. al.^31^ with the addition of an elastic structure around the fluidic oscillator being the most prominent difference. In our setup, a laminar incompressible fluid flow is considered and the elastic material is isotropic with linear elasticity. See the “Methods” section for a detailed explanation of the simulation setup. Two observables were measured to evaluate the oscillatory characteristics. The first one is the pressure loss coefficient ${C}_{P}$, which correlates to the energy loss required to maintain the oscillation. A smaller value of ${C}_{P}$ corresponds to better energy efficiency. The second one is the driving force coefficient ${C}_{D}$, which correlates to the force driving the flow to the feedback channels. Hence, a larger value of ${C}_{D}$ suggests a larger lateral momentum inside the mixing chamber of the fluidic oscillator. These two observables are measured as

1 $$\begin{array}{c}{C}_{P}=2\left({p}_{2}-{p}_{1}\right),\;\;{\text{and}}\;\;{C}_{D}=\cfrac{2\left({p}_{4}-{p}_{3}\right){d}_{i}}{d}, \end{array}$$

where ${p}_{1}$ – ${p}_{4}$are the dimensionless pressure values measured at four specific locations in the computational model, ${d}_{\rm{i}}$ is the power nozzle width, and $d$ is the feedback channel width, as shown in Fig. 3. The time series for both observables are shown in Fig. 3a. It is clear that ${C}_{P}$ is oscillating twice as fast as ${C}_{D}$ because the sweeping fluid motion passes through the outlet twice in a single sweep. The time course of ${C}_{D}$ is obtained by measuring the pressure difference in the lateral direction, corresponding to the oscillatory characteristics of the lateral sweeping motion inside the fluidic oscillator, which is of our interest. Hence, the time course of ${C}_{D}$ will be used as the basis for the phase reduction analysis.

Since the addition of the elastic structure is intended to give controllability of the oscillatory dynamics, the intrinsic oscillatory dynamics inside the fluidic oscillator is expected to be similar to a conventional fluidic oscillator with no moving parts when there is no periodic forcing applied to the elastic structure. To confirm this, we compare the resulting Strouhal number $St$ for a given Reynolds number $Re$ with and without the addition of the elastic structures on the design. We confirm that the addition of the elastic structures itself does not affect the intrinsic oscillatory dynamics of the fluid flow inside the fluidic oscillator, as shown in Fig. 3b (see “Methods” section for details). Since the Strouhal number can be considered as the normalized oscillation frequency with respect to the fluid velocity, Fig. 3b shows that the oscillation frequency scales linearly with the Reynolds number, which is an expected characteristic for fluidic oscillators^7,32^. For the remaining sections that follow, all computations are conducted for $Re=350$.

### Phase reduction analysis

**Figure 4:**
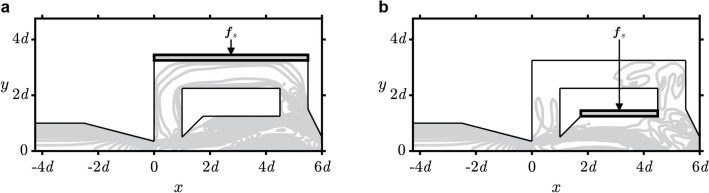
Placement of the elastic structures. (**a**) First configuration: attached around the feedback channel. (**b**) Second configuration: attached around the mixing chamber. ${{\varvec{f}}}_{\rm{s}}$ represents the external forcing applied to the elastic structure boundary, and the arrow represents the location where it is applied.

In this part, we show how dimensionality reduction is achieved by employing phase reduction analysis, which reduces the theoretically infinite-dimensional multiphysics system into a single scalar phase equation. We first consider two configurations of the elastic structure placement. The first configuration has the elastic structures attached around the feedback channels and the second one has the structures attached around the mixing chamber, as shown in Fig. 4a, b respectively. For each condition, we use what is known as the direct method^24,33^ to evaluate the phase sensitivity function $Z$ of the system by applying a localized impulse perturbation ${{\varvec{f}}}_{s}=\varepsilon \delta \left({\varvec{x}}-{{\varvec{x}}}_{0}\right)\delta \left(t-{t}_{0}\right)\left({-\widehat{{\varvec{e}}}}_{y}\right)$, where $\varepsilon \ll 1$ is the amplitude, $\delta \left({\varvec{x}}-{{\varvec{x}}}_{0}\right)$ represents a gaussian impulse function in spatial coordinates with $\delta \left(t-{t}_{0}\right)$ being its temporal domain counterpart, and ${\widehat{{\varvec{e}}}}_{y}$ is a unit vector in the $y$-axis direction. The location ${{\varvec{x}}}_{0}$ in which these perturbations are applied, is shown in both Fig. 4a, b, and is imposed at multiple phase values determined by ${t}_{0}$. Refer to the “Methods” section on how the phase sensitivity function is evaluated after the impulse perturbations are applied.

**Figure 5:**
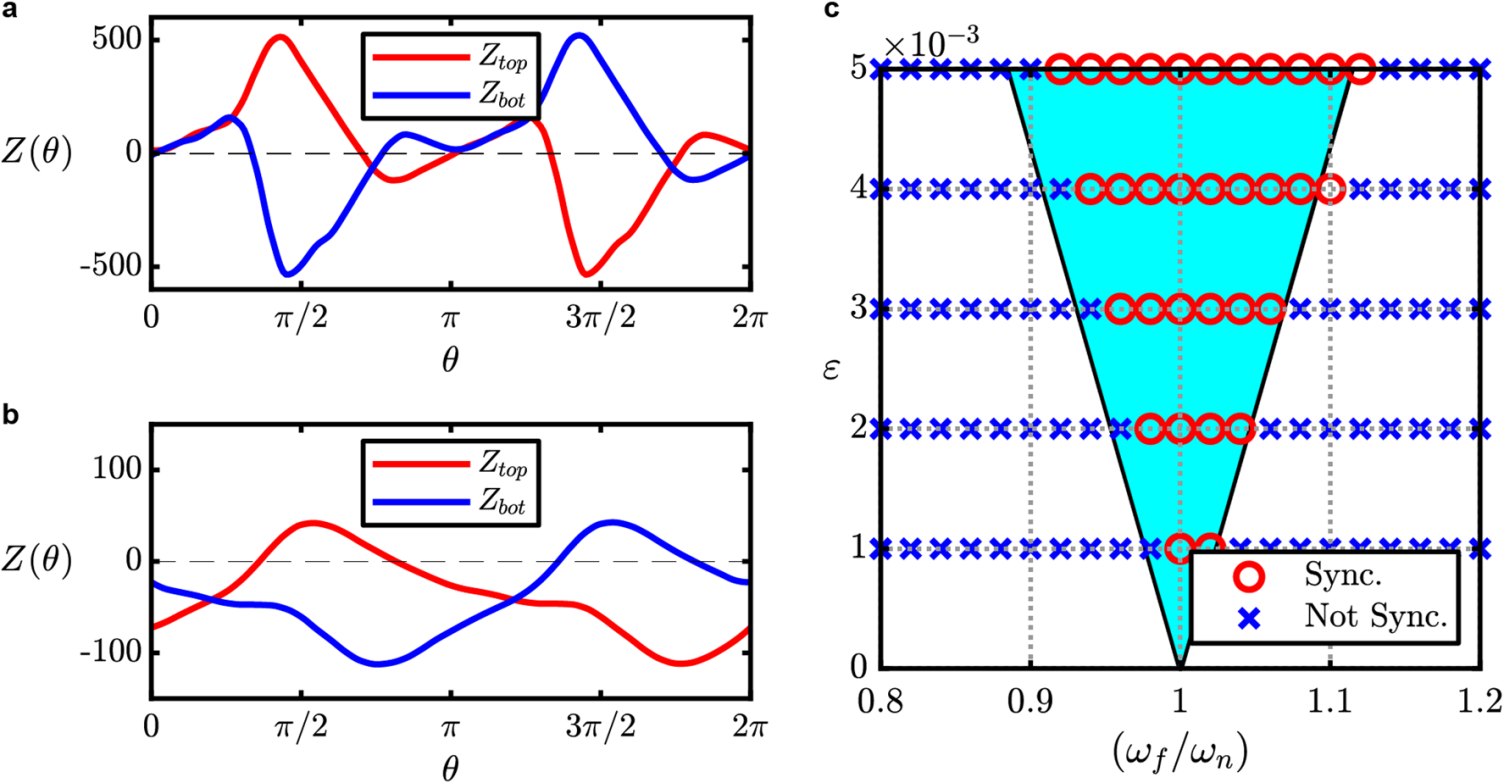
Results for phase sensitivity and synchronization analysis. (**a**) Phase sensitivity function evaluated when the elastic structure is attached around the feedback channels. (**b**) The same as (**a**), but when elastic structure is attached around the mixing chamber. (**c**) The Arnold tongue^36^ (covered within the triangular region) showing the estimated synchronization region, and its comparison with actual results from direct numerical simulations.

The phase sensitivity functions have larger peak-to-peak values for the first configuration compared to the second one, as shown in Fig. 5a, b, respectively. This implies that the system is more sensitive to external perturbation applied around the feedback channels rather than those applied around the mixing chamber. Hence, we can expect a wider synchronization region with respect to the amplitude and frequency of the external periodic forcing for the first configuration. In a more general context, these findings also show how placement of the external forcing around the feedback region of a system will cause a larger change in the system behavior. While the feedback region is easily identified for the case of the feedback-type fluidic oscillator, it is not always easy to identify the corresponding region for other kinds of oscillatory flows, such as for the case of vortex shedding^1,24^ or feedback-free fluidic oscillator^34^.

It is also found that there is a symmetrical property between the phase sensitivity functions of the top and bottom sides where ${Z}_{top}\left(\theta ,-{\widehat{{\varvec{e}}}}_{y}\right)={Z}_{bot}\left(\theta +\pi ,{\widehat{{\varvec{e}}}}_{y}\right)$. This is expected given the spatially symmetric property of the oscillatory flow inside the fluidic oscillator and the phase difference introduced by the alternating sweeping motion. This finding is consistent with other studies of oscillatory flows with symmetrical properties^35^. This symmetrical property also implies that the largest synchronization region with respect to the external forcing frequency and amplitude is achieved when anti-symmetric forcing is applied to the system with a 1:1 synchronization. In other words, when the ratio of the external forcing frequency ${\omega }_{f}$ and the original oscillation frequency ${\omega }_{n}$ is approximately equal to 1. On the other hand, for symmetric forcing, it can be inferred that a larger synchronization region is likely to be achieved for with 1:2 synchronization (${{\omega }_{f}/\omega }_{n}\approx 2$)^21,23^.

### Synchronization analysis

In this section, we validate the phase equation obtained from phase reduction analysis, and analyze the effects of periodic external perturbations on the oscillatory characteristics. From here onwards, we consider only the first configuration as shown in Fig. 4a. We analyze the phase dynamics of the system given that a periodic perturbation is applied in the form of a simple sinusoidal function. Utilizing the symmetrical property, we apply anti-symmetric forcing to the outer boundaries of the elastic structures in the following form

2 $${{\varvec{f}}}_{\rm{s}}=\left\{\begin{array}{c}\begin{array}{ll}\cfrac{\varepsilon }{2}\delta \left({\varvec{x}}-{{\varvec{x}}}_{0}\right)\mathrm{sin}\left({\omega }_{f}\left(t-{t}_{0}\right)\right)\left({-\widehat{{\varvec{e}}}}_{y}\right)& ,\;\text{for the top side,}\\ \cfrac{\varepsilon }{2}\delta \left({\varvec{x}}-{{\varvec{x}}}_{0}\right)\mathrm{sin}\left({\omega }_{f}\left(t-{t}_{0}\right)+\pi \right)\left({\widehat{{\varvec{e}}}}_{y}\right)& ,\;\text{for the bottom side.}\end{array}\end{array}\right.$$

Using phase reduction theory, we can determine the synchronization region based on the external forcing frequency and amplitude, also known as Arnold Tongue^36^. From the results obtained with direct numerical simulations (DNS), synchronization is determined where the phase difference between the original oscillation and the external forcing converges to a constant value. To validate the synchronization region, multiple simulations were conducted using parameters ${0.8\le {\omega }_{f}/\omega }_{n}\le 1.2$ with step size of $0.02$, and $0.001\le \varepsilon \le 0.005$ with step size of $0.001$. The estimated synchronization region evaluated using phase reduction theory is in good agreement with results from DNS, as shown in Fig. 5c.

**Figure 6:**
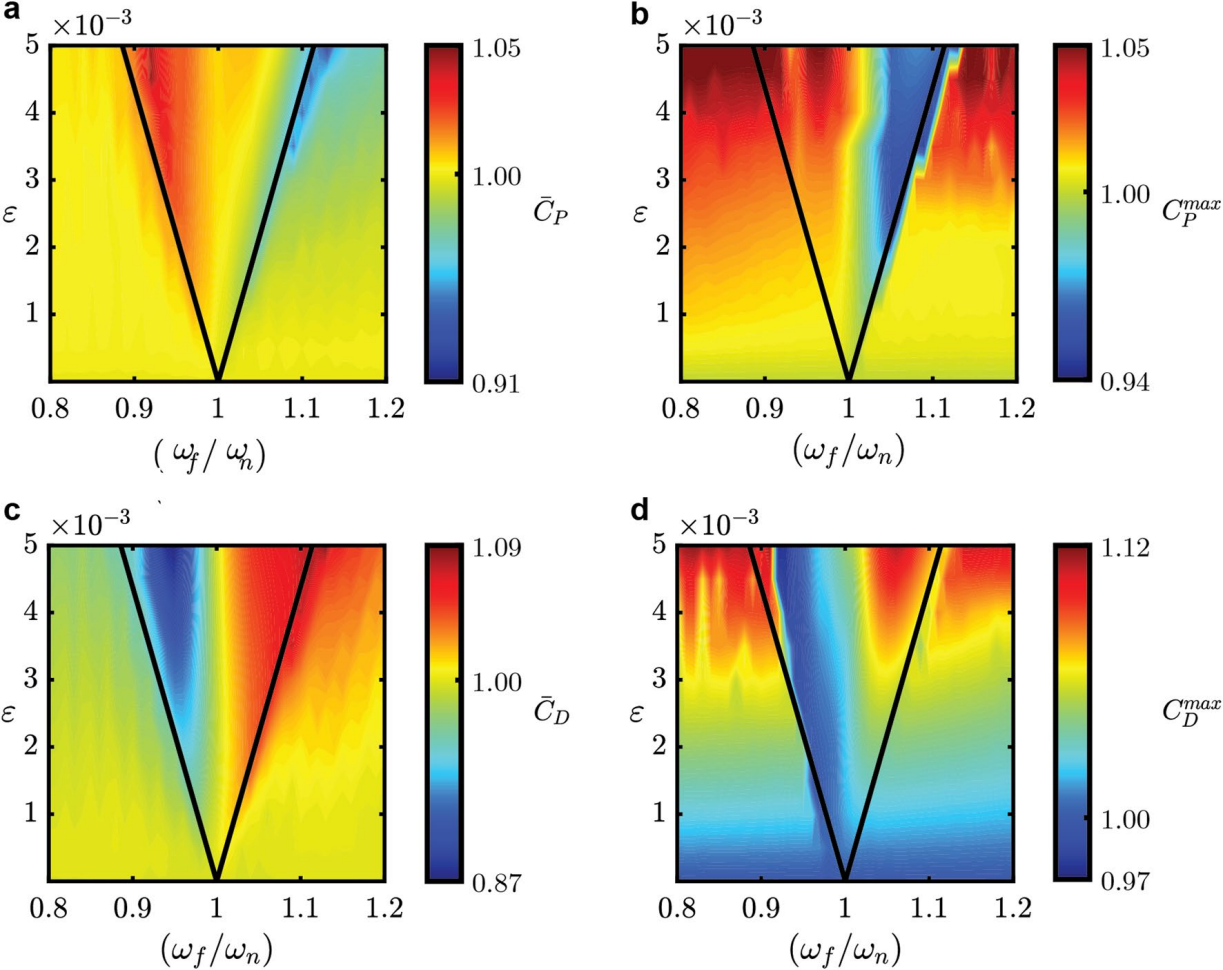
Ratio of several fluid flow properties inside the fluidic oscillator due to periodic forcing with their nominal values. The Arnold tongue is illustrated by the triangular region in the middle of each figure. (**a**) RMS value of the pressure loss coefficient. (**b**) Maximum value of the pressure loss coefficient. (**c**) RMS value of the driving force coefficient. (**d**) Maximum value of the driving force coefficient.

We further analyze the change in the physical characteristics of the oscillatory flow due to the periodic forcing. This is done by comparing the ratio of the root-mean-square (RMS) values and the maximum values of both ${C}_{P}$ and ${C}_{D}$ after the periodic forcing is applied with their nominal values when no external forcing is present. For the synchronizing cases, the RMS and maximum values can be evaluated after one period of oscillation once synchronization is observed. For the non-synchronizing cases, the phase difference will increase indefinitely^33,37^, so the said values are evaluated after at least one cycle of phase slip^33^ is observed. The results are displayed in Fig. 6, with ${\overline{C}}_{P}$ and ${\overline{C}}_{D}$ denoting the ratio of RMS values and ${C}_{P}^{max}$ and ${C}_{D}^{max}$ denoting the ratio of maximum values. The parameters used are the same as the ones used for validating the synchronization region, and a smooth colormap is obtained with interpolation in-between the data points. For the synchronizing cases where ${\omega }_{f}/{\omega }_{n}>1$, it can be inferred that the overall internal fluid velocity also increases given that the oscillatory flow frequency increases. However, Fig. 6a, b, show that both the RMS and maximum values of ${C}_{P}$ decrease with increasing frequency ratio, which is somewhat contradictory with the conventional case where a larger outlet to inlet pressure difference is required to increase the flow velocity. We propose that this observation is obtained because the vibrating elastic structure imposes additional momentum into the fluid flow, such that a faster sweeping motion is maintained even without an increase in the inlet pressure. Although an increase in energy efficiency is seen in the fluid flow perspective, an additional energy source is required to actuate the elastic structure vibration. Even so, our proposed method avoids the need to increase the inlet pressure to increase the oscillation frequency. It is also worth noting that for non-synchronizing cases, the maximum value of ${C}_{P}$ increases. As mentioned, this can be undesirable since the material used to make the device needs to withstand a larger inlet pressure value^12^. This exemplifies the importance of estimating the synchronization region using phase reduction theory.

We can also infer from the results that an increase of momentum in the lateral direction around the mixing chamber is observed for the synchronizing cases where ${\omega }_{f}/{\omega }_{n}>1$, as shown by the increase of both the RMS and maximum values of ${C}_{D}$ in Fig. 6c, d. The increase in oscillation frequency is a desirable effect for fluid mixing applications, since it is known that a higher oscillation frequency improves the mixing efficiency^7,38^. Since in the current study we consider an incompressible fluid such that its density does not change, the increase in lateral momentum indicates that there is an increase in the lateral fluid velocity, which promotes convective mixing^39^. We also note that the increase in the RMS value of ${C}_{D}$ is more apparent in the synchronizing cases compared to the non-synchronizing cases for the same value of periodic forcing amplitude where ${\omega }_{f}/{\omega }_{n}>1$. We can infer that a larger increase in the internal fluid velocity is achieved with a smaller forcing amplitude when synchronization is achieved. This further shows the advantage of estimating the synchronization region in advance and reduces the number of parametric studies required to find the optimal forcing amplitude and frequency to reach the desired system characteristics.

We have shown that by using phase reduction theory, the phase response of the given fluidic oscillator design is estimated well, and that synchronization with external forcing imposed by elastic structure vibration changes the internal fluid flow properties. This can be utilized in practical applications such as fluid mixing. Studies on mixing of chemical substances have considered the use of fluidic oscillators with many shapes and forms^7,11^. However, the resulting behavior is largely determined by the geometry of the device. The method proposed in this study introduces added controllability to the mixing behavior while retaining the overall geometry of the device. Moreover, the dimensionality reduction using phase reduction theory presented in this study might prove useful for other studies using fluidic oscillators, such as synchronization analysis of cascaded fluidic oscillators and controllability analysis by means of a different perturbation method^9,15,16^.

While several recent works have involved rigorous mathematical work to obtain analytical solutions for the phase equation of oscillatory flows^40,41^, we acknowledge that phase reduction alone does not allow estimation of the changes in the fluid flow properties due to synchronization. For instance, direct numerical simulations still need to be done for multiple values of periodic forcing frequencies and amplitudes before the changes in fluid flow properties can be analyzed, such as values of ${C}_{P}$ and ${C}_{D}$ as shown in Fig. 6. Phase-amplitude reduction^42,43^, which is a well-known extension of the phase reduction method, is a promising solution to this problem since it allows prediction of the future state variables such as the fluid flow properties. Although a sophisticated method of phase-amplitude reduction has been proposed recently^44^, direct implementation and confirmation of its validity for spatio-temporal dynamics in multiphysics system, including fluid–structure coupled dynamics, still needs to be confirmed.

### Optimal control

The phase reduction analysis allows us to predict the synchronization condition. However, there is no constraint regarding the time required to reach the synchronization. Depending on the amplitude and shape of the periodic forcing signal, it can take multiple periods of oscillation before synchronization is achieved. This is why an optimal control framework^37,45,46^ is desired with the goal to design a periodic forcing signal (i.e. the control signal) that achieves synchronization within one period of oscillation for a given averaged power.

**Figure 7:**
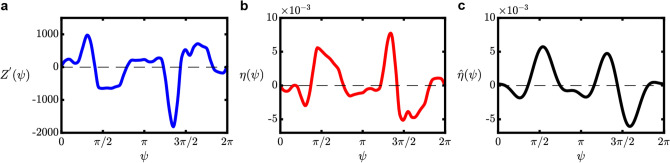
Design of the optimal periodic forcing signal. (**a**) The gradient of phase sensitivity function (evaluated numerically). (**b**) The optimal control signal. (**c**) The reduced form of the optimal control signal.

The optimal control signal can be designed given that the phase sensitivity function $Z$, the phase difference during synchronization $\Delta \omega$, and its average power $P$ are known, such that

3 $$\begin{array}{c}\displaystyle{\eta }_{\varepsilon }\left(\psi \right)=\frac{1}{2\lambda }{Z}^{^{\prime}}\left(\psi \right)-\Delta \omega \frac{Z\left(\psi \right)}{\langle {Z}^{2}\rangle },\;\;{\text{and}}\;\;\lambda =-\frac{1}{2}\sqrt{\frac{\langle {\left({Z}^{^{\prime}}\right)}^{2}\rangle }{P-\cfrac{{\left(\Delta \omega \right)}^{2}}{\langle {Z}^{2}\rangle }}},\end{array}$$

where ${\eta }_{\varepsilon }\left(\psi \right)$ is the optimal periodic forcing signal with amplitude $\varepsilon$, ${Z}^{^{\prime}}\left(\psi \right)=dZ\left(\psi \right)/d\psi$ is the gradient of the phase sensitivity function along the periodic phase value $\psi =[0, 2\pi )$, which can be evaluated numerically if $Z$ is known, and $\lambda$ is the Lagrange multiplier introduced for the optimization problem, and $\Delta \omega ={\omega }_{n}-{\omega }_{f}$. Explanation for Eq. (3) is described in the “Methods” section. The evaluated ${Z}^{^{\prime}}\left(\psi \right)$ in this study is shown in Fig. 7a.

To demonstrate this idea, we choose a test case where a pure sinusoidal periodic perturbation with amplitude $\varepsilon =0.004$ and frequency is applied to the system. When synchronization is achieved, the frequency difference is found at $\Delta \omega =-0.06$ and the calculated average power of the periodic forcing signal is $P=8\times {10}^{-6}$. Given these values, we can construct the optimal control signal based on Eq. (3), as shown in Fig. 7b. The optimal control signal has a complex shape, since it contains many harmonic components. This is a consequence of the framework where ${\eta }_{\varepsilon }\left(\psi \right)$ is essentially a scaled version of ${Z}^{^{\prime}}\left(\psi \right)$, which contains many harmonic components in this case. However, judging from the amplitudes, each harmonic components exhibit only a small amount of power. Hence, we can adopt a reduced version of ${\eta }_{\varepsilon }\left(\psi \right)$ by decomposing it into its Fourier series components such that

4 $$\begin{array}{c}\eta {}_{\varepsilon }\left(\psi \right)\cong {\sum }_{k=0}^{N}\;{a}_{k}\;\mathrm{cos}\left(k\psi +{b}_{k}\right), \end{array}$$

where ${a}_{k}$ and ${b}_{k}$ are the amplitude and phase shift of each $k$-th harmonic component respectively, and $N$ represents the minimum number of harmonic components required to approximately represent the signal in Fourier series relative to its average power. For instance, we consider a constraint that 80% of the average power is required to reach optimal synchronization time. We can construct a control signal $\widehat{\eta }{}_{\varepsilon }\left(\psi \right)$ by choosing the $M$ components of the Fourier series representation of $\eta {}_{\varepsilon }\left(\psi \right)$ that satisfies

5 $$\begin{array}{c}\widehat{\eta }{}_{\varepsilon }\left(\psi \right)\cong {\sum }_{k=0}^{M}\;{a}_{k}\;\mathrm{cos}\left(k\psi +{b}_{k}\right),\;\;{\text{where}}\;\;{\sum }_{k}^{M}{\Vert {a}_{k}\Vert }^{2}\ge0.8\;{\sum }_{k}^{N}{\Vert {a}_{k}\Vert }^{2}, \end{array}$$

The resulting control signal after this reduction is shown in Fig. 7c, where the periodic forcing signal become less complex in shape, indicating that a practical implementation is more plausible.

**Figure 8:**
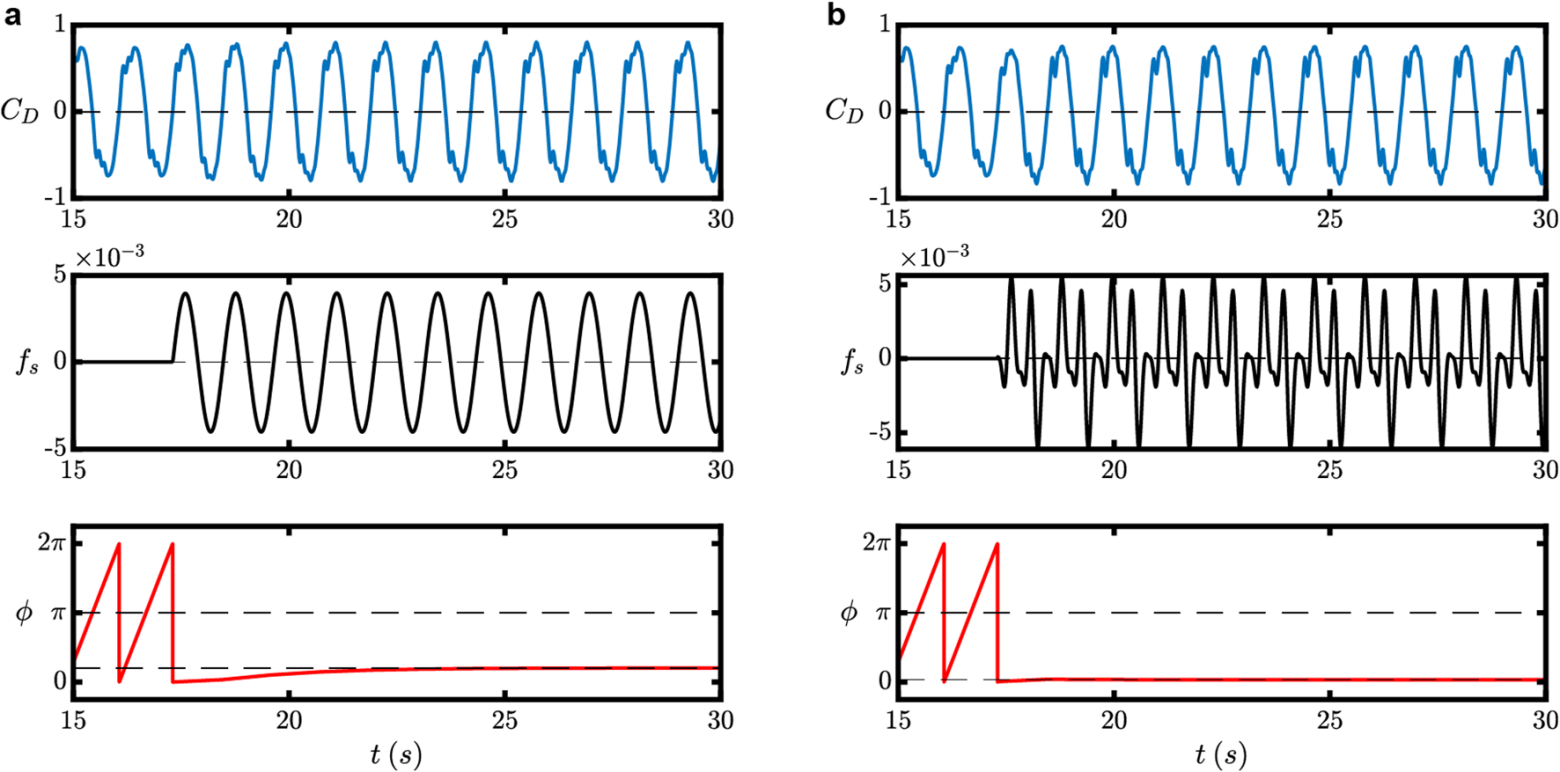
Comparison of time to synchronization depending on the periodic forcing signal. (**a**) Pure sinusoidal forcing tooks around 5 periods of oscillation before synchronization is achieved. (**b**) Even with a reduced version of the optimal control signal, one period of oscillation is enough to achieve synchronization.

We conduct direct numerical simulation to compare the time required for synchronization for two different cases, where the periodic forcing applied to the system is either a purely sinusoidal signal or the reduced version of the optimal control signal. Both periodic forcing satisfies $\Delta \omega =-0.06$ and $P=8\times {10}^{-6}$ as described previously in the design process of the optimal control signal. Figure 8a shows that it takes around 5 periods of oscillation to reach synchronization for a purely sinusoidal forcing, while Fig. 8b shows that the reduced version of the optimal control signal only requires one period of oscillation. We also note that there is no significant difference with the amplitude response of the driving force coefficient ${C}_{D}$ for both cases.

In general, when a dynamical system with a large degree-of-freedom is reduced to a phase equation, the resulting phase sensitivity function will have a complex waveform^47^ due to the existence of higher-order harmonic terms. This also applies to the fluid–structure coupled system in the present study. Here, we proposed a method to optimize the transition time to the synchronized state by designing a smoother waveform of the external forcing and demonstrated its effectiveness. The proposed method can reach a synchronized state with optimal transition time, even for actual experimental systems where a complex periodic waveform cannot be applied due to physical constraints.

## Conclusions

In this work, we have introduced a design concept of a feedback-type fluidic oscillator surrounded by elastic structures. Our approach maintains the intrinsic oscillatory flow characteristics inside the fluidic oscillator but introduces controllability through synchronization with weak external forcing from the moving component. The resulting changes in physical characteristics due to synchronization can be advantageous for practical applications such as fluid mixing, without any need to change the overall geometry or to increase the inlet pressure. We demonstrate how the synchronization condition can be estimated well using phase reduction theory, even for a complex fluid–structure coupled system, allowing for the desired system characteristics to be discovered more efficiently. Furthermore, taking practical applications into account, we demonstrate how synchronization can be achieved within one period of oscillation by implementation of the optimal control framework.

## Methods

### Computational model

In this study, numerical simulations for the two-dimensional model of the fluidic oscillator based on the finite element method (FEM) are conducted using commercial software COMSOL Multiphysics^48^ version 5.5. The fluid flow is governed by the incompressible Navier–Stokes equation, with the dimensionless conservation of mass and momentum equation in Arbitrary Lagragian–Eulerian formulation as follows

6 $$\begin{array}{c}\sqrt{\cfrac{M}{{C}_{Y}}}\cfrac{\partial {{\varvec{u}}}_{f}}{\partial t}+\left({{\varvec{u}}}_{f}-{{\varvec{u}}}_{m}\right)\cdot \nabla {{\varvec{u}}}_{f}=\nabla \cdot \left\{-p\mathbf{I}+\cfrac{1}{Re}\left[\nabla {{\varvec{u}}}_{f}+{\left(\nabla {{\varvec{u}}}_{f}\right)}^{\mathrm{T}}\right]\right\},\;\;{\text{and}}\;\;{\nabla \cdot {\varvec{u}}}_{f}=0,\end{array}$$

where ${{\varvec{u}}}_{f}$ is the fluid flow velocity, ${{\varvec{u}}}_{m}$ is the spatial coordinate velocity, $p$ is the pressure, $M$ is the fluid-to-structure density ratio, and ${C}_{Y}$ is the Cauchy number, $\mathbf{I}$ is the identity matrix, and external forcing such as gravity are neglected. The structural mechanics dimensionless equation is

7 $$\begin{array}{c}\cfrac{{\partial }^{2}{{\varvec{w}}}_{s}}{\partial {t}^{2}}+\alpha \cfrac{\partial {{\varvec{w}}}_{s}}{\partial t}=\nabla \cdot \left({{{\varvec{\upsigma}}}_{s}}^{\mathrm{T}}+\beta \cfrac{\partial {{{\varvec{\upsigma}}}_{s}}^{\mathrm{T}}}{\partial t}\right)+{{\varvec{f}}}_{s} ,\end{array}$$

where ${{\varvec{w}}}_{s}$ is the structural displacement, ${{\varvec{\sigma}}}_{s}$ is the stress tensor, $\alpha$ is the mass damping coefficient, $\beta$ is the stiffness damping coefficient, and ${{\varvec{f}}}_{s}$ is the external forcing. At the fluid–structure interface, the dimensionless equations for the kinematic and dynamic coupling conditions are

8 $$\begin{array}{c}{{\varvec{u}}}_{f}=D\sqrt{\cfrac{\mathcal{M}}{{C}_{Y}} }\cfrac{{\partial {\varvec{w}}}_{s}}{\partial t},\;\;{\text{and}}\;\; {{\varvec{\sigma}}}_{s}{\cdot {\varvec{e}}}_{n}={C}_{Y}\left\{-p\mathbf{I}+\cfrac{1}{Re}\left[\nabla {{\varvec{u}}}_{f}+{\left(\nabla {{\varvec{u}}}_{f}\right)}^{\mathrm{T}}\right]\right\}{\cdot {\varvec{e}}}_{n}, \end{array}$$

where $D$ is the displacement coefficient, ${{\varvec{e}}}_{n}$ is a unit vector normal to the fluid–structure interface. In our setup, the dimensionless parameters are set such that $M=1.27\times {10}^{-4}$, ${C}_{Y}=2.50\times {10}^{-6}$, $Re=350$, $\alpha =1.5\times {10}^{-3}$, $\beta =175$, and $D=1.81\times {10}^{-3}$.

The geometry of the computational model is shown in Fig. 2 where its dimensions are normalized to the width of the feedback channel $d$. The power nozzle width is ${d}_{i}=0.7d$. The elastic structure on each side has a thickness of $0.2d$. A parabolic velocity profile ${U}_{in}=6U(y+1)\left(H-(y+1)\right)/{H}^{2}$ is set for the fluid inlet boundary condition, where $U$ is the mean velocity and $H=2d$ is the inlet channel width. The fluid outlet boundary condition is set to a static pressure ${p}_{\rm{out}}=0$. The left and right boundaries of the elastic structures are fixed, that is ${\partial {\varvec{w}}}_{s}/\partial t=0$. The computational domain is filled with quadrilateral meshes that are symmetrical with respect to the line $y=0$.

### Phase reduction and synchronization analysis

We briefly describe the formulation for phase reduction analysis as follows. Consider the system state of the FSI dynamics ${\varvec{X}}({\varvec{x}},t)={\left[{{\varvec{u}}}_{\rm{f}}({\varvec{x}},t), {{\varvec{w}}}_{\rm{s}}({\varvec{x}},t)\right]}^{\mathrm{T}}$ and the state dynamics when an external forcing is applied as

9 $$\begin{array}{c}\cfrac{\partial }{\partial t}\varvec{X}\left({\varvec{x}},t\right)=\varvec{F}\left\{{\varvec{X}}\left({\varvec{x}},t\right)\right\}+\varepsilon\varvec{\eta} \left({\varvec{x}},t\right), \end{array}$$

where ${\varvec{x}}=\left(x,y\right)$ is the coordinates in which measurements are taken that is theoretically infinite, ${\varvec{F}}\left\{\cdot \right\}$ is a functional representing the state dynamics, $\varepsilon$ is the perturbation amplitude and ${\varvec{\eta}}\left({\varvec{x}},t\right)$ is the perturbation function. The system exhibits a stable limit-cycle oscillation when no perturbation is applied $\left(\varepsilon =0\right)$, such that ${\varvec{X}}\left({\varvec{x}},t\right)={\varvec{X}}\left({\varvec{x}},t+2\pi /{\omega }_{n}\right)$. By introducing a phase functional ${\varvec{\Theta}}\left\{{\varvec{X}}\left({\varvec{x}},t\right)\right\}$, the system state can be mapped into scalar phase values such that $\theta \left(t\right)={\varvec{\Theta}}\left\{{\varvec{X}}\left({\varvec{x}},t\right)\right\}$, where $\theta \in \left[\mathrm{0,2}\pi \right]$. The phase functional is defined in such a way that $\theta$ increases with a constant frequency ${\omega }_{n}$, that is

10 $$\begin{array}{c}\cfrac{d\theta }{dt}={\omega }_{n}=\displaystyle\int \left(\frac{\delta{\varvec{\Theta}}}{\delta {\varvec{X}}}\right)\cdot {\varvec{F}}\left\{{\varvec{X}}\left({\varvec{x}},t\right)\right\}d\varvec{x}, \end{array}$$

where ${\varvec{Z}}\left(\theta ;{\varvec{x}}\right)=\delta{\varvec{\Theta}}/\delta {\varvec{X}}$ is the phase sensitivity function, which can be approximated along the limit-cycle orbit. Should an external forcing with amplitude $0<\varepsilon \ll 1$ be applied, the phase dynamics now becomes

11 $$\begin{array}{c}\cfrac{d\theta }{dt}={\omega }_{n}+\varepsilon\displaystyle\int {\varvec{Z}}\left(\theta ;{\varvec{x}}\right)\cdot{\varvec{\eta}}\left({\varvec{x}},t\right)d\varvec{x}. \end{array}$$

Given this formulation, we can utilize impulse perturbation method, also known as the direct method^24,33^, in order to evaluate ${\varvec{Z}}\left(\theta ;{\varvec{x}}\right)$. We consider the case in which a weak and localized impulse forcing is applied, that is $\varepsilon \ll 1$ and $\int{\varvec{\eta}}\left({\varvec{x}},t\right)d{\varvec{x}}=\delta \left(t-{t}_{0}\right)$, where $\delta$ is a gaussian impulse function with small enough standard deviation. In this study, we achieve this by setting ${\varvec{\eta}}\left({\varvec{x}},t\right)=\delta \left({\varvec{x}}-{{\varvec{x}}}_{0}\right)\delta \left(t-{t}_{0}\right)\left(-{\widehat{{\varvec{e}}}}_{y}\right)$. The gaussian impulse function $\delta \left({\varvec{x}}-{{\varvec{x}}}_{0}\right)$ has a standard deviation of $0.1d$, while $\delta \left(t-{t}_{0}\right)$ has a standard deviation of $0.02T$, where $T$ is the oscillation period for ${C}_{D}$. By applying the impulse perturbation, a phase shift asymptotic to the original limit cycle orbit ${\varvec{g}}\left(\theta ;{\varvec{x}}, \varepsilon \right)$ will be introduced. We can then evaluate the phase sensitivity function by the following approximation.

12 $$\begin{array}{c}\varvec{Z}\left(\theta ;{\varvec{x}}\right)=\underset{\varepsilon \to 0}{\mathrm{lim}}\cfrac{{\varvec{g}}\left(\theta ;{\varvec{x}}, \varepsilon \right)}{\varepsilon }\approx \cfrac{{\varvec{g}}\left(\theta ;{\varvec{x}}, \varepsilon \right)}{\varepsilon }. \end{array}$$

Moving on, we will consider the case where the perturbation is localized in the spatial coordinate, such that ${\varvec{x}}$ can be dropped from the notation. We applied the impulse perturbation at 20 different phase values within a single oscillation (determined by ${t}_{0}$) in order to obtain the phase sensitivity function $Z$, as shown in Fig. 5a, b. The synchronization condition is achieved if the phase difference between the oscillatory system and the periodic forcing $\phi \left(t\right)=\theta \left(t\right)-\psi \left(t\right)$ converges to a constant value (phase-locked), where $\psi \left(t\right)={\omega }_{f}t$. In other words, $d\phi /dt=\Delta \omega +\varepsilon\Gamma \left(\phi \right)$ converges to 0, where $\Delta \omega ={\omega }_{n}-{\omega }_{f}$ and $\Gamma \left(\phi \right)$ is the phase coupling function defined as

13 $$\begin{array}{c}\displaystyle\Gamma \left(\phi \right)=\frac{1}{2\pi }\int_{0}^{2\pi }Z\left(\phi +\psi \right)\cdot \eta \left(\frac{\psi }{{\omega }_{f}}\right)d\psi , \end{array}$$

which is a $2\pi$ periodic function that is evaluated using averaging approximation due to the fact that $\phi \left(t\right)$ is changing slowly over time compared to $\psi \left(t\right)$. With these formulations, it is possible to approximate the synchronization condition based on the forcing amplitude and frequency, such that

14 $$\begin{array}{l}\varepsilon\;{\mathrm{ min}}\;\Gamma \left(\phi \right)<-\Delta \omega <\varepsilon\;{\mathrm{ max}}\;\Gamma \left(\phi \right). \end{array}$$

### Optimal control framework

Recall that synchronization is achieved when the oscillatory system and the periodic forcing are phase-locked. By this definition, a stable fixed point ${\phi }_{s}^{*}$ must exist, such that $d\phi /dt=\Delta \omega +{\Gamma }_{\varepsilon }\left(\phi \right)$ converges to 0 at $\phi ={\phi }_{s}^{*}$, where ${\Gamma }_{\varepsilon }\left(\phi \right)=\varepsilon\Gamma \left(\phi \right)$ for simplicity. We can infer that in order to achieve the fastest convergence, we need to have the steepest gradient possible for ${\Gamma }_{\varepsilon }\left(\phi \right)$ around $\phi ={\phi }_{s}^{*}$. In other words, the goal of optimal control is to

15 $$\begin{array}{l}{\text{maximize}}\;-{\Gamma }_{\varepsilon }^{{\prime}}\left({\phi }_{s}^{*}\right)=-\cfrac{d}{d\phi }{\Gamma }_{\varepsilon }\left({\phi }_{s}^{*}\right). \end{array}$$

This goal must be combined with the following phase-locking constraint and the averaged power constraint

16 $$\begin{array}{l}\cfrac{d\phi }{dt}=\Delta \omega +{\Gamma }_{\varepsilon }\left({\phi }_{s}^{*}\right)=0,\;\;{\text{and}}\;\;P=\langle {\eta }_{\varepsilon }^{2}\rangle \end{array}$$

where $P$ is the averaged power exhibited by the periodic forcing signal ${\eta }_{\varepsilon }\left(\psi \right)$ with amplitude $\varepsilon$, and $\langle \cdot \rangle$ denotes the averaging operator. Through these goals and constraints, we can construct the following cost function of the optimization problem

17 $$\begin{array}{ll}C\left\{\eta \left(\psi \right)\right\}&{}=-{\Gamma }_{\varepsilon }^{^{\prime}}\left({\phi }_{s}^{*}\right)+\lambda \left(\langle {\eta }_{\varepsilon }^{2}\rangle -P\right)+\gamma \left(\Delta \omega +{\Gamma }_{\varepsilon }\left({\phi }_{s}^{*}\right)\right)\\&{}=\displaystyle\frac{1}{2\pi }\int_{0}^{2\pi }\left\{{\eta }_{\varepsilon }\left(\psi \right)\left[\gamma Z\left(\psi +{\phi }_{s}^{*}\right)-{Z}^{^{\prime}}\left(\psi +{\phi }_{s}^{*}\right)+\lambda {\eta }_{\varepsilon }\left(\psi \right)\right]-\lambda P+\gamma\Delta \omega \right\}d\psi , \end{array}$$

Furthermore, by using calculus of variations, the optimal periodic forcing signal can be proven to be equal to Eq. (3). Equation (3) also shows that the optimal periodic forcing signal can be designed given that the phase sensitivity function $Z$, the phase difference during synchronization $\Delta \omega$, and its averaged power $P$ are known. In practice, we can initially figure out $Z$ using the phase reduction analysis and use a purely sinusoidal periodic forcing signal to figure out $P$ which is required to reach synchronization with a particular $\Delta \omega$ based on the synchronization condition shown in Eq. (14). These values can then be substituted back to Eq. (3) to evaluate the optimal periodic forcing signal ${\eta }_{\varepsilon }\left(\psi \right)$.

## Data Availability

The experimental dataset that supports the findings of this study is available from the corresponding author upon reasonable request.
